# Effect of Heat Stress on Lactating and Non-Lactating Blackbelly Ewes under Tropical Conditions during Summer

**DOI:** 10.3390/ani14132003

**Published:** 2024-07-07

**Authors:** Edgar Valencia-Franco, Ethel Caterina García y González, Aurora Matilde Guevara-Arroyo, Fernando Torres-Agatón, José Manuel Robles-Robles, José del Carmen Rodríguez-Castillo, Marisol Paredes-Alvarado, Luis Alaniz-Gutiérrez, Maricela Ruiz-Ortega, José Luis Ponce-Covarrubias

**Affiliations:** 1 Escuela Superior de Medicina Veterinaria y Zootecnia No. 3, Universidad Autónoma de Guerrero (UAGro), Técpan de Galeana 40900, Guerrero, Mexico; edgar.valencia@correo.buap.mx (E.V.-F.); 17905@uagro.mx (E.C.G.y.-G.); 09972@uagro.mx (A.M.G.-A.); 14173@uagro.mx (F.T.-A.); 2 Facultad de Ciencias Agrícolas y Pecuarias, Benemérita Universidad Autónoma de Puebla, Tlatlauquitepec 73900, Puebla, Mexico; 3 Facultad de Medicina Veterinaria y Zootecnia, Benemérita Universidad Autónoma de Puebla, El Salado, Tecamachalco 72570, Puebla, Mexico; manuel.roblesr@correo.buap.mx (J.M.R.-R.); jose.rodriguez@correo.buap.mx (J.d.C.R.-C.); 4 Facultad de Estudios Superiores Cuautitlán, Universidad Nacional Autónoma de México (UNAM), Cuautitlán Izcalli 54714, Edo de México, Mexico; paredesmapa@cuautitlan.unam.mx; 5 Facultad de Medicina Veterinaria y Zootecnia No. 2, Universidad Autónoma de Guerrero, Cuajinicuilapa 41940, Guerrero, Mexico; alanizl@uagro.mx; 6 Instituto de Ciencias Agropecuarias, Universidad Autónoma del Estado de Hidalgo, Tulancingo de Bravo 43600, Hidalgo, Mexico

**Keywords:** greenhouse effect, climate change, environmental temperature, RH, thermoregulation, skin temperatures, hair ewes

## Abstract

**Simple Summary:**

Currently, climate change and greenhouse emissions are a phenomenon that threatens the production of foods of animal origin and food security. This has been observed in agroecological regions where heat stress did not exist and, in regions where temperatures and relative humidity were high, it is intensifying. Hair sheep show resistance to heat stress (HS) in different warm latitudes; however, lactating ewes, due to metabolic work, produce a greater amount of heat and have more problems dissipating heat than those ewes that do not lactate. To counteract the effects caused by HS, ewes adjust their physiological parameters to avoid productive and reproductive problems that affect their production and well-being.

**Abstract:**

Two groups of ewes (10 lactating and 10 non-lactating) were used to evaluate the effect of heat stress during summer under tropical conditions. In this study, a temperature and humidity index (THI) was found that ranged between 65 and 79 (morning and afternoon). Likewise, a heat tolerance coefficient (HTC) of 6 units was observed. The highest breathing frequency (BF; 115.46 ± 35.25 breaths per minute (bpm)) and rectal temperature (RT; 38.95 ± 0.51 °C) were found during the afternoon in the group of lactating ewes. The means were compared by group, time of the day, and interaction, and only significant differences were found between groups for RT and udder temperature (*p* < 0.001). In the case of time of day, all parameters were higher during the afternoon, regardless of the group of ewes (*p* < 0.001). Likewise, an interaction was found in the parameters RT, right paralumbar fossa (RPF), rump, leg, and udder (*p* < 0.001). In conclusion, Blackbelly ewes lactating during the summer in the tropics have higher skin temperatures, and also raise BF and RT to tolerate HS in tropical climates.

## 1. Introduction

Worldwide, sheep production predominates in arid, semi-arid, desert, and tropical regions. These regions are characterized by being hostile and hot, and have climates that cause heat stress in production animals [1,2]. Heat stress (HS) is the sum of external environmental sources that affect the animal, causing an increase in rectal temperature (RT), and activating some physiological parameters and behavioral heat regulation [3].

Mexico has various agroecological regions (arid to tropical), with the tropics representing 30% of the national territory [4]. In the Mexican tropics, in the south of the country, high environmental temperatures (35 °C) and relative humidity (75%) represent a challenge for heat-stressed ewes [4,5,6].

HS is the result of a combination of climatic factors, high environmental temperature, humidity, solar radiation, and wind speed, with negative impacts on health, welfare, and animal production [5]. Problems are observed in different domestic production species, especially in dairy cattle (see review: [7]) and pigs (see review: [8]), which are the animals most sensitive to the effect of heat because of the thermoregulation problems due to the metabolic work that these species perform. HS affects the reproduction and production of ewes (meat and milk), significantly increasing this effect as global temperatures increase [9,10]. These characteristics are observed in arid [11] and tropical [5,6] regions of Mexico, where in addition to production being compromised, damage is observed in the behavior and health of sheep. The HS in ewes changes according to the physiological stage of the females, i.e., the heat load is influenced depending on whether ewes are pregnant, empty, or lactating. For example, it has been observed that pregnant ewes show higher BF and RT than empty ewes [12,13]. Lactating ewes, due to the metabolic work they do to maintain milk production, increase their body temperature compared to ewes that are not lactating [10,13]. In fact, it has been shown that lactating ewes maintain plasma levels of prolactin to maintain lactation, an event that causes greater heat loads due to milk production [10,14].

In tropical regions, there are few studies evaluating thermoregulation in lactating hair ewes. Lactation is a key biological stage for the metabolic activity of the female, reflecting an increased production of physiological heat, which together with ambient temperature is expressed in thermoregulatory problems [13,15]. An increase in BF, RT, and blood thyroid hormone profiles has been reported in lactating ewes under HS conditions [16]. Given these alterations according to age and lactation, some data indicate that lactating ewes are more sensitive to HS, due to problems in thermoregulation [11,16]. In another study, an increase in BF and RT was reported in lactating ewes that produced a greater amount of milk compared to those with lower production [14]. Therefore, the aim of the present study was to evaluate the effect of heat stress during the summer in lactating vs. non-lactating Blackbelly ewes under tropical conditions.

## 2. Materials and Methods

### 2.1. General

This study was carried out in June and July of 2021 at the Sheep and Goat Zootechnical post of ESMVZ-3, UAGro. The institution is located in the municipality of Tecpan de Galeana, Guerrero, Mexico (17°13′21″ N 100°37′53″ W). The maximum ambient temperature is 40 °C, during the summer, and the minimum temperature is 17 °C, during the winter months [4]. All animal management procedures were conducted within the guidelines of nationally approved techniques for animal use and care [17]. The experimental protocol was approved on 20 February 2020 by the Committee for the Uses and Care of Experimental Animals of the Autonomous University of Guerrero (protocol #1009).

### 2.2. Animals and Treatments

The experimental period lasted 31 days. A total of 20 multiparous Blackbelly ewes were divided into groups (lactating and not lactating; 10 ewes/group). At the start of the experiment, the lactating ewes had an average weight of 31.6 ± 6.54 kg and that of non-lactating ewes was 35.09 ± 2.68 kg, with a body condition score of 2.17 ± 0.55 and 2.67 ± 0.59 Pt BCS (5-point scale, with increments between units of 0.25; 1 = emaciated and 5 = obese) [18] (Table 1).

### 2.3. Weather Conditions

The ambient temperature (°C) and relative humidity (%) were recorded using electronic sensors connected directly to the meteorological station that is monitored by the national meteorological system, located in the municipality of Tecpan de Galeana, Guerrero, Mexico (# 12233, 17°15′00″ N, 100°34′07″ W, altitude 262.0 MSNM [4]. The environmental parameters were measured for 31 days every third day in the months of June and July 2021. The meteorological station is located 2 km from the location where the study was located; temperature and humidity recording was carried out every third day from 6:00 to 18:00 h.

The level of HS was evaluated through the indices that were calculated in the following manner:

1. Humidity and temperature index: this was calculated using Kibler’s calculation (1964) [19]: THI= 1.8 AT − (1 − RH) (AT − 14.3) + 32, where AT is the average temperature (°C) and RH is the relative humidity. This index is only 79, indicating an alert condition, detected with the signal from the World Meteorological Organization [20].
2. Heat tolerance coefficient (HTC): the modification proposed by López et al. [21] was used, for ewes, using the formula: HTC = (RT/39) + (BF/23), where RT is rectal temperature (°C) and BF is breathing frequency (inspirations/min) [22]. This coefficient indicates that when HTC is close to two, the physiological parameters of the animals indicate that there is no HS or that the animal is tolerant to heat. However, when HTC is >2, it indicates that there is HS.

### 2.4. Physiological Parameters and Skin Temperatures 

In this study, physiological parameters ((breathing frequency (BF) and rectal temperature (RT)) and skin temperatures (head, neck, scapula, right para-lumbar fossa (RPF), abdomen, haunch, leg, udder, and vulva) were measured in each ewe [5]. BF was measured by counting the number of movements per minute of the RPF and RT was measured with a digital thermometer (Delta Trak^®^, Pleasanton, CA, USA). To measure skin temperatures, full-body photographs were taken with a thermal imaging camera (Fluke Ti10, Everett, WA, USA), subsequently viewed on a computer with Fluke Smart View^®^ 3.9 software. All parameters were recorded at two hours of the day (07:00 and 15:00 h).

### 2.5. Food and Accommodation

The group of experimental ewes was confined in a pen (13.20 m long and 13.80 m wide) during the evaluation period. The pen was equipped with feeders and waterers on a galley with a galvanized sheet in the center of the corral (0.96 m wide and 0.68 m high). All the ewes were grazed in two periods, from 8:00 to 12:00 h, and again from 17:00 to 19:00 h. During grazing, they consumed guinea grass (*Panicum maximum*), grass *(Cynodon dactylon*), and dry leaves of trees. The pen always had clean, fresh water ad libitum for the ewes to drink upon returning from grazing.

### 2.6. Statistical Analysis

The parameters were analyzed using an ANOVA that presents descriptive statistics, considering the behavior of the parameters within and between groups (lactating and non-lactating). The evaluated parameters are presented as means ± standard deviation. The data were validated in relation to the group of ewes (lactating and non-lactating) and the time of day (morning and afternoon), and the interaction between group and time of day, using repeated measurements under a General Linear Model (PROC GLM). A comparison test of means was performed using a “Tukey” test at a significance level of 0.05. All data were analyzed with the SAS v. 9.0 statistical package [23]. 

## 3. Results

### 3.1. Weather Conditions

**Figure 1:**
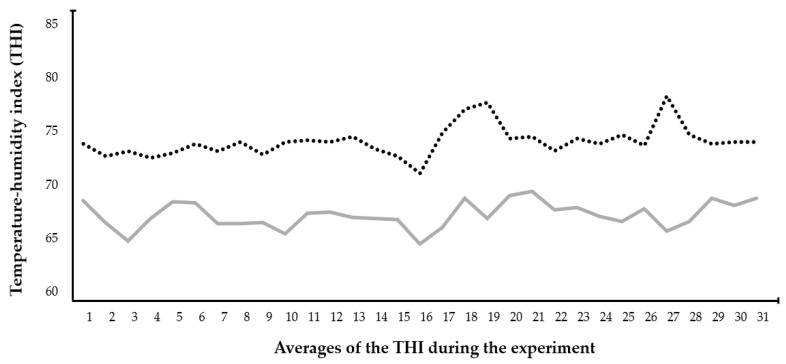
Temperature and humidity index (THI) values during the afternoon (dotted line) and morning (continuous line), during the experimental period.

THI results ranged from 69 to 79; values of 68.05 ± 1.223 were obtained during the morning. Values of 74.902 ± 1.422 were obtained during the afternoon, with a minimum of 71.932 and a maximum of 79.127 (Figure 1).

### 3.2. Heat Tolerance Coefficient and Physiological Parameters

According to the publication by Lopez et al. [21], when the HCT exceeds 2 units it is considered a heat stress environment. In the present study, the ewes of the lactating group exceeded 4 units, and in the case of the average records during the afternoon it reached 6 units, indicating severe heat stress for the ewes (Table 2).

Regardless of the experimental group, during the afternoon the average was higher than 5.8 units, classified as severe HS. Although during the morning the average did not reach 3 units, it was considered to be environmental conditions that cause HS in ewes (Table 3).

In the study, the highest BF occurred during the afternoon in the group of lactating ewes, of 115.46 ± 35.25 bpm. The same phenomenon was recorded for RT during the afternoon in lactating (38.95 ± 0.51 °C) and non-lactating (38.93 ± 0.56 °C) ewes (Table 3). The temperature of the vulva during the afternoon in the group of lactating ewes was higher (38.63 ± 20.05 °C) than in the group of non-lactating ewes (37.48 ± 1.27 °C) (Table 4). 

### 3.3. Skin Temperatures

Statistical results showed that all skin temperatures were significantly higher in the afternoon compared to the morning, regardless of the group (*p* < 0001) (Table 5).

A comparison of means was performed by group (lactating vs. non-lactating) and by time of day (morning vs. afternoon) and interaction (group × time of day). In the case of group, significant differences were found only between the group for the variable RT and udder temperature (*p* < 0.001). In the case of time of day, all parameters were greater during the afternoon, regardless of the group of ewes (*p* < 0.001). In the case of the interaction, a significant difference was found in the parameters RT, RPF, rump, leg, and udder (*p* < 0.001) (Table 6). 

## 4. Discussion

In the present study, environmental conditions indicative of HS for sheep in the tropics of Guerrero, Mexico were measured. Indeed, a high THI (65 to 79) and a heat tolerance coefficient of 6 units were found, indicating that the degree of HS is severe. Likewise, the physiological parameters (BF and RT) and skin temperatures (head, neck, scapula, RPF, abdomen, haunch, leg, udder, and vulva) were higher during the afternoon in the group of ewes lactating than during the morning. The above demonstrates the relationship that exists between the physiological response of ewes under HS environmental conditions. 

### 4.1. Weather Conditions

The thermoneutral zone for hair sheep is between 15 and 30 °C and a THI < 74, which are classified as an absence of HS [24]. In the present study, the maximum temperatures were 38.8 °C and the THI was 79, demonstrating that the ewes were under severe HS. For hair breed sheep, it is considered that animal welfare and HS are in conflict when the THI is ≥72, and BF and RT are increased to maintain the comfort zone [25]. For example, sheep that remain in environments with HS increase the values of BF, RT, RH, and skin temperatures, altering their physiological functions [26]. When RH is low, it favors heat exchange through respiratory or skin evaporation, maintaining the comfort of the ewes [27]. This causes problems such as dryness of the mucous membranes and difficulty in the exchange of heat through non-evaporative (conduction, radiation, and convection) and evaporative (sweating and respiration) mechanisms, hindering the thermoregulation of these females [26].

Results similar to those of the present study were reported by Seixas et al. [24] under tropical conditions in northwest Brazil (monthly temperature, 31.6 to 35.5 °C, and THI, 76.8 to 80.4), demonstrating that the ewes were in HS conditions. However, the above intensifies according to the physiological stage of the females. In this regard, García y González et al. [6] found HS in lambs and multiparous ewes intensified in the afternoon. In the present study, we worked with multiparous lactating and non-lactating ewes; although HS was found in both groups of females, in the lactating group it was more intense. In this regard, Macías-Cruz et al. [11] found higher RT and BF during the afternoon in lactating ewes, and the same phenomenon occurred for the skin temperatures presented by these ewes. 

In hot climates, lactating females can increase energy requirements for maintenance by between 7% and 25%, and, by inducing an increase in RT and BF, food consumption in HS ewes is decreased. Therefore, it is important to provide processed foods to reduce metabolic work and heat load. Fat and nitrogen reserves are used to provide energy through glucose biosynthesis at the expense of the active mammary gland [28]. The ewes used in this study are not milk producers, but the milk they produce is sufficient to increase metabolic heat, which, when added to ambient heat and food intake, indicates an effect of HS [16].

Some studies have reported that when the HTC indicator is 2 U, sheep do not present HS [21,22], and when it is increased to 3 U, the RT increases by 0.5 °C. In the present experiment, HTC was found during the morning to be 2.4 to 2.6 U, and during the afternoon it exceeded 6 U, which confirms that ewes from the Guerrero tropics suffer severe HS regardless of the group. However, this is more intense in lactating ewes than in non-lactating ewes. Consistent with these results, Serrano-Torres et al. [25] and Souza et al. [22], under tropical conditions, found HTC values similar to those reported in the present study. In another study, Mehaba et al. [10] found higher RT and BF in lactating ewes under HS conditions. Likewise, García y González et al. [13] found an increase in physiological parameters and skin temperatures in heat-stressed ewes during summer in the tropics. 

### 4.2. Heat Tolerance Coefficient and Physiological Parameters 

To maintain their productivity and, in some cases, guarantee their survival, sheep need to maintain their body temperature within the physiological limits for the species, that is, their homeothermy [26]. The physiological parameters (BF and RT) are indicators of the animal’s degree of comfort and it is ability to tolerate environmental conditions [5,29]. The normal BF for sheep in the thermoneutral zone is 25 to 61 bpm [30]. On the other hand, in severe HS conditions, it can rise to 300 or 400 bpm [5]. Environmental temperature plays an important role in the increase in these parameters; for example, when environmental temperatures are above 35 °C, ewes increase heat loss through the respiratory tract by up to 60% [31]. However, the increase in muscle activity due to the respiratory process can be disadvantageous due to the considerable amount of heat generated by the work performed by respiratory muscles [32]. In this regard, Saldaña-Ríos et al. [29] found a high BF in heat-stressed Dorper (116 ± 32 bpm), Katahdin (109 ± 38 bpm), and Pelibuey (86 ± 36 rpm) breed sheep in a humid tropical climate. 

In the present study, lactating ewes maintained average BF during the morning of 37.7 bpm and in the afternoon of 115.4 bpm, which was higher than the ewes that were not lactating. This is logical since lactating ewes, due to the production of metabolic heat, try to dissipate heat through respiration. Indeed, in heat-stressed ewes, a decrease in BF by 20% was observed at midday, but in the afternoon, it increased to 66% [11]. 

This adaptation mechanism that hair ewes present to reduce body water losses and avoid dehydration in conditions of HS was observed in both groups of ewes [5], since during the afternoon the increase in BF was 68% in the ewes of the present study.

In the case of RT, in sheep the normal average is 39° C and it ranges between 38 and 40 °C [30]. The temperature obtained in the study by Saldaña-Ríos et al. [29] was higher than 38.7 °C, which is similar to that reported by Quesada et al. [31] (38.8 °C) in ewes under HS conditions. This same phenomenon was found in ewes of the Dorper (39 ± 0.45 °C), Katahdin (39 ± 0.37 °C), and Pelibuey (39 ± 0.66 °C) breeds under humid tropical conditions [29]. In the present study, non-lactating ewes presented a higher RT at both times of the day, in the morning with 38 °C and in the afternoon with an average of 38.9 °C. In this regard, Ruiz-Ortega et al. [5] suggests that an increase in RT demonstrates that the heat release mechanisms are not efficient. In this sense, Saldaña-Ríos et al. [29] observed in climates with an average ambient temperature of 38.5 °C and 80% RH, a maximum average RT of 41.6 °C, specifying that the range was outside normality for ewes. Therefore, the thermoregulation mechanisms were not sufficient, since the high percentage of RH, environmental temperature, and solar radiation favor HS, decreasing the gradient of body heat dissipation. Macías-Cruz et al. [11] published changes in RT, BF, and skin temperatures in the summer, correlated with changes in environmental temperature and THI throughout the day. This same phenomenon was published in regions of the Guerrero tropics in multiparous ewes and lambs under HS during the summer [5,6]. Other investigations agreed by obtaining similar results in their experiments, where the physiological parameters increased with the increase in environmental temperature, both under HS and thermoneutrality conditions [24,27,33].

### 4.3. Skin Temperatures

The exchange of heat between the body and the environment through the skin is also considered important in the thermoregulation of ewes in climates that cause HS [27,34]. A skin temperature higher than the ambient temperature causes a release of body heat, but when it is lower it causes a gain in environmental heat [34]. When measuring the surface temperature on the back, hip, side, forehead, and base of the skull, a direct effect of solar radiation on the surface located towards the dorsal region has been reported; compared to the forelimb and hindlimb, together, in the rostral region a selective cooling mechanism of the brain has been reported in sheep [35], associated with greater activity of the superficial veins to maintain brain temperature below the average body temperature [32].

In an experiment conducted in northwest Mexico in desert climates, temperatures of different body areas were highest (*p* < 0.05) at midday and lowest (*p* < 0.05) between midnight and mornings during the summer [11]. However, the temperature of the head was not affected (*p* > 0.05) at midnight or in the morning, but the temperatures of the right paralumbar fossa, scapula, and haunch were higher (*p* < 0.05). In the case of surface temperatures, all body regions were higher (*p* < 0.05) at midday [11]. In this same work, the skin temperatures (scapula, haunch, and right paralumbar fossa) showed that, during the month of August when compared with the other summer months, the dissipation of body heat was greater during the mornings, in the afternoon there was a gain in heat, and at midnight the body and environmental temperatures balanced. In another study carried out in sheep under tropical conditions, Santos et al. [36] reported body temperatures of 39–39.2 °C in the morning and 39.6–39.7 °C in the afternoon. 

In the present investigation, lactating ewes presented higher surface temperature values in the afternoon; however, significant differences were found by group. The temperature was higher in the afternoon in the lactating group in the head (36.6 °C), neck (36.5 °C), scapula (36 °C), abdomen (37.2 °C), udder (35.4 °C), leg (37.3 °C), and vulva (38.6 °C). In the case of the right paralumbar fossa zone and the rump, it was higher for the group of non-lactating ewes (36.7 °C) than the lactating group (35.9 °C) in the afternoon. Some research suggests that the values of the thermal gradient do not present a significant effect (*p* > 0.05) in the hours of 1:00 p.m. and 2:00 p.m., but a significant effect (*p* < 0.05) has been observed at 3:00 p.m., where RT, environmental temperature, and skin temperatures have higher values [26]. Furthermore, it is observed that, at 3:00 p.m., the gradient between RT and surface temperature is low, so it is difficult for the sheep to eliminate excess body heat. This phenomenon can be explained by the presence of the short wool layer on its body [11]. Recent studies corroborate that BF, RT, and THI are predictors of HS in ewes [6]. Under tropical conditions, the recording of skin temperatures allows a rapid evaluation of HS conditions [32]. The study by Reyes et al. [32] indicates that by taking the skin temperatures on the back (≥41.2 °C), hip (≥39.0 °C), and side (38.2 °C), the RT can be ≥39.6 °C. In this sense, in another study carried out in an arid region, they found higher skin temperatures in ewes in the morning, noon, and afternoon during the month of August, which is when the summer heat is most intense; but they also observed greater dissipation of body heat through the scapula, haunch, and right paralumbar fossa [11]. The high correlations found between BF, RT, and temperature in different body parts of ewes with the THI indicate that they are excellent predictors of HS [25]. Most of the thermoregulation in heat-stressed ewes occurs through the respiratory tract (60%), and the rest occurs through the skin, regardless of the physiological stage. 

Currently, due to the phenomenon of global warming, production animals suffer from HS [2]; therefore, it is important that the general population becomes aware of the care of the environment and animals. The current environmental conditions result in serious consequences for production (meat and milk), health, animal welfare, and the economy of farmers [11]. Faced with this challenge, it is necessary to implement teaching strategies in veterinary medicine study programs to train students and new veterinarians so that they can face this problem, which is becoming more serious every day [37,38]. Researchers must consider one of the basic axes of the concept of animal protection in their research: the principle of the three Rs, i.e., replacement, reduction, and refinement [37]. This will allow the number of animals to be reduced, especially in HS work with ewes, to avoid the manipulation of many animals during the experimental phase, which, combined with the stress caused by the environment, could increase the animals’ stress [5]. Finally, it is necessary to advise producers to implement HS mitigation strategies considering the physiological stage (empty, pregnant, and lactating ewes) [2]. Therefore, the triad of education of students, farmers, and researchers will contribute to implementing research, management, and production strategies for species of zootechnical interest in the face of the growing environmental threat to the production of protein of animal origin.

## 5. Conclusions

It is concluded that lactating Blackbelly ewes undergo severe HS during the summer, and this was observed due to the high degree of THI, in addition to the fact that they present high skin temperatures and increased BF and RT. Lactating ewes have a higher HS. This is because the accelerated metabolism produces greater heat and, consequently, in females with hyperthermia, they take time to dissipate it due to the humidity present in the environment. It is necessary to search for strategies to mitigate HS in females in this physiological stage and prevent the effects caused by these conditions.

## Figures and Tables

**Table 1:** Live weight and body condition score of the experimental groups.

Group	Variable	Average ± SD	Minimum	Maximum
Lactating	LW (Kg)	35.09 ± 2.68	30	41.3
	BCS (Pt)	2.17 ± 0.55	1	3.5
Not lactating	LW (Kg)	31.6 ± 6.54	23.7	45.1
	BCS (Pt)	2.67 ± 0.59	1.5	4

Standard deviation (SD), minimum (min), maximum (max), live weight (LW), body condition score (BCS), and points (Pt).

**Table 2:** Average HTC of the ewes regardless of the group.

Ewes Group	HTC
Lactating	4.3 (average of all lactating ewes regardless of time).
Non-lactating	4.1 (average of all non-lactating ewes regardless of time).
Measurement time	
Morning	2.4 (average of all morning values regardless of group).
Afternoon	6 (average of all afternoon values regardless of group).

**Table 3:** Heat tolerance coefficient (HTC) by time of day in lactating and non-lactating ewes.

Group	Time of the Day	N	Average ± SD
Lactating	Morning	288	2.603 ± 0.613
	Afternoon	288	6.019 ± 1.539
Not lactating	Morning	288	2.467 ± 0.417
	Afternoon	288	5.881 ± 1.642

Standard deviation (SD).

**Table 4:** Descriptive data of the BF (bpm) and RT (°C) of lactating and non-lactating ewes.

Variable	Lactating				Non-Lactating			
	Morning		Afternoon				Afternoon	
	BF	RT	BF	RT	BF	RT	BF	RT
Average	37.72	37.56	115.46	38.95	34.3	38.06	112.31	38.93
Standard deviation	14.02	0.71	35.25	0.51	9.52	1.01	37.66	0.56
Minimum	20	28.9	28	34.2	20	27	24	32.2
Maximum	116	38.8	168	40.2	92	40.9	168	40.9

Number of observations (N = 288), standard deviation (SD).

**Table 5:** Mean and standard deviation (SD) at different body temperatures (°C) in lactating and non-lactating ewes.

Variable	Lactating		Non-Lactating		*p*-Value
	Morning	Afternoon	Morning	Afternoon	
Head	29.97 ± 1.62	36.56 ± 17.76	29.45 ± 1.8	36.17 ± 2.2	0.3942
Neck	31.3 ± 1.75	36.77 ± 17.76	30.25 ± 11.92	35.63 ± 2.01	0.0839
Scapula	32.26 ± 1.4	36.07 ± 1.79	31.02 ± 11.67	35.65 ± 1.86	0.0188
RPF	31.09 ± 2.38	35.82 ± 1.96	28.81 ± 2.12	35.91 ± 2.37	<0.0001
Abdomen	33.75 ± 1.52	37.17 ± 1.54	32.85 ± 16.3	36.58 ± 1.81	0.123
Haunch	30.35 ± 11.86	35.36 ± 2.25	28.21 ± 1.91	36.74 ± 19.79	0.5803
Leg	31.53 ± 14.4	37.32 ± 1.44	32.63 ± 1.57	36.76 ± 1.50	<0.0001
Udder	34.19 ± 1.39	35.47 ± 1.93	28.66 ± 2.01	35.2 ± 2.11	0.0003
Vulva	34.24 ± 1.65	38.63 ± 20.05	34.07 ± 1.73	37.48 ± 1.27	0.2703

*p*-Value corresponds to purchasing between groups.

**Table 6:** *p* values for the effects of group, time of day, and their interaction.

Variable	Group	Time of the Day	Interaction
BF	0.0404	<0.0001	0.9317
RT	<0.0001	<0.0001	<0.0001
HTC	0.050	<0.0001	0.9920
Head	0.3942	<0.0001	0.9071
Neck	0.0839	<0.0001	0.9384
Scapula	0.0188	<0.0001	0.251
RPF	<0.0001	<0.0001	<0.0001
Abdomen	0.123	<0.0001	0.7488
Haunch	0.5803	<0.0001	0.0103
Leg	0.0003	<0.0001	0.0031
Udder	<0.0001	<0.0001	<0.0001
Vulva	0.2703	<0.0001	0.4115

## Data Availability

The authors declare that all data used in the research will be available and without access restrictions to those who request it.
